# Laryngotracheal Abnormalities in Esophageal Atresia Patients: A Hidden Entity

**DOI:** 10.3389/fped.2018.00401

**Published:** 2018-12-18

**Authors:** Andrea Conforti, Laura Valfrè, Marianna Scuglia, Marilena Trozzi, Duino Meucci, Stefania Sgrò, Sergio Bottero, Pietro Bagolan

**Affiliations:** ^1^Neonatal Surgery Unit, Department of Medical and Surgical Neonatology, Bambino Gesù Children's Hospital, Rome, Italy; ^2^Airway Surgery Unit, Department of Pediatric Surgery, Bambino Gesù Children's Hospital, Rome, Italy; ^3^Anesthesiology Unit, Department of Anesthesiology, Bambino Gesù Children's Hospital, Rome, Italy

**Keywords:** esophageal atresia, esophageal atresia and tracheoesophageal fistula, laryngotracheal anomalies, airways abnormalieties, laryngobronchoscopy, trachea abnormalities, tracheal surgery, subglottic stenosis of the trachea

## Abstract

**Importance:** Presence of laryngotracheal abnormalities is associated with increased morbidity and higher mortality rate in esophageal atresia patients.

**Objective:** Determine the prevalence of laryngotracheal abnormalities (LTA) in a prospectively collected cohort of patients treated for esophageal atresia and/or tracheoesophageal fistula (EA/TEF). Analysis of the impact of those airway anomalies in early post-operative outcomes was performed.

**Patients and Methods:** This was a review of a prospectively collected database, including patients from January 2008 to December 2017. Patients enrolled in the present study were treated in a high-volume referral center. Present study included all newborn-infants consecutively treated for EA/TEF. All patients were evaluated by flexible laryngotracheoscopy performed under local anesthesia in spontaneous breathing. In case of airway malformation suspected during flexible endoscopy, a rigid endoscopy was performed to complete airway assessment. If post-operative respiratory symptoms (noisy breathing, respiratory difficulty, failure to extubate, or difficulty feeding) were noted, a second laryngotracheoscopy was performed. Primary study outcome was to evaluate the prevalence of LTA in EA/TEF infants, characterizing of LTA, and their impact on early post-operative outcomes. Those primary study outcomes were planned before data collection began.

**Results:** During the study period 207 patients with EA/TEF were treated. LTA had a period prevalence of 40.1% (83/207). Although no differences were recorded in terms of demographics and clinical presentation, LTA+ infants more frequently required tracheostomy (12/52, 23% vs. 0/124, 0%; *p* 0.0001) and were at increased risk of death (12/83, 14% vs. 5/124, 4%; *p* 0.009) in comparison with EA/TEF without LTA.

**Conclusions:** Present data suggest a high prevalence of congenital LTA in patients affected by EA. Most of the abnormalities are congenital and a high proportion of patients with LTA require a tracheostomy. Mortality significantly correlates with the presence of LTA. Systematic airway endoscopic preoperative evaluation has to be pushed forward to minimize LTA-related morbidity and mortality.

## Introduction

Over the past several decades, advances in surgical techniques and perioperative neonatal care have led to low mortality rates in patients with esophageal atresia with/without tracheoesophageal fistula (EA/TEF) (1). Therefore, late morbidities have become an important issue during the follow-up of these children.

A recent paper from EUROCAT Working Group reported the presence of associated abnormalities in more than 50% of patients with EA/TEF (2): interestingly, some of those associated abnormalities may have great clinical implication, with the potential to modify surgical plan and medical management (3). Associated anomalies can be chromosomal (Trisomy 21, trisomy 18), part of a genetic syndrome (CHARGE syndrome, Feingold syndrome and others), associations such as VACTERL-sequence or multiple anomalies without a pattern. To date, the most common associated anomalies are congenital heart defects (described in about 30% of all patients), urinary tract and limb anomalies (in more than 15% and 13% of EA/TEF infants, respectively), and other gastrointestinal anomalies (reported in about 15% of patients) (2, 4). Less explored is the association with laryngo-tracheal abnormalities.

In a retrospective selected group of EA/TEF patients, the Boston group reported the frequent association (up to 37.5%) between EA/TEF and upper airway conditions (e.g., vocal fold paralysis, laryngeal cleft, and tracheomalacia) (5). Those patients were diagnosed following esophageal surgery when noisy breathing, respiratory difficulties, failure to extubate, or difficulty feeding were complained by patients or their families. Similarly, a recent report from a French and Canadian collaborative group, reported retrospectively a prevalence of LTA in about 30% AE/TEF patients (6). Therefore, nowadays, one of the major concerns regarding this association is the frequent post-discharge diagnosis of upper airways malformation, leading to increased morbidity and even mortality.

Ideally these associate conditions should be ruled out during the first admission for EA/TEF, to modulate surgical planning and follow-up, reducing surgical challenge, parental stress, and possible adverse consequences linked to an unknown malformation.

Therefore, aim of the present study was to prospectively evaluate the prevalence of airway abnormalities, detected before esophageal surgery, in a high flow referral center of newborn/infants affected by EA/TEF, also analyzing the impact of LTA in early post-operative outcomes.

## Patients and Methods

This is a review of a prospectively collected database. The Bambino Gesù Children's Scientific Institutional Review Board (201602P003688) approved it waiving the need for informed consent because of the observational nature of the study.

All newborn-infants admitted for EA/TEF between January 2008 and December 2016 were enrolled into the present study. Confirmation of EA/TEF was performed in all patients via preoperative tracheobroncoscopy (p-TBS) as part of standardized approach. All infants were then operated on by dedicated team of neonatal surgeons, and all were treated according to our published algorithm for assessment and management of EA/TEF patients (3).

The cornerstones of our algorithm are:

1. Preoperative EA classification based on Gross classification (7) via p-TBS. Flexible awake bronchoscopy is aimed to evaluate preoperative vocal cord motility, and tracheomalacia (maintaining spontaneous breathing), while defining the presence of unexpected proximal fistula.
2. Preoperative gap measurement, reported in detail previously (3), performed at time of p-TBS in all babies with any type of EA/TEF. Long-gap esophageal atresia (LGEA) was defined as EA with no distal fistula, any type of EA with a preoperative esophageal gap ≥3 vertebral bodies or patients referred with cervical esophagostomy after a previous failed attempt elsewhere.
3. Preoperative assessment of specific laryngo-tracheal associated anomalies.

Focusing on the first aim (evaluate the prevalence of airway abnormalities), specific attention was dedicated to detection and classification of associated LTA, to evaluate their prevalence in our EA/TEF population. All patients were evaluated by pediatric ENT team with flexible laryngotracheoscopy performed under topical anesthesia in spontaneous breathing. In case of airway malformation suspected during flexible endoscopy, a rigid endoscopy was performed to complete airway assessment. In cases with post-operative respiratory symptoms (noisy breathing, respiratory difficulty, failure to extubate, or difficulty feeding) a second laryngo-tracheo-bronchoscopy was performed in the operative theater.

LTA were defined as presence of moderate to severe tracheomalacia according to Benjamin (8), AND/OR grade of subglottic stenosis (9) AND/OR vocal cord paralysis (mono or bilateral), AND/OR other major LTA (cleft, major complex anomalies).

In order to evaluate the role of LTA in early post-operative outcomes, patients were grouped based on LTA presence/absence: Group A comprised 83 patients presenting LTA in addition to EA/TEF, while Group B included 124 infants without LTA. The outcomes considered were age at definitive esophageal surgery, rate of primary anastomosis, need for special technique or refinements to achieve esophageal elongation, re-do esophageal surgery, need for tracheostomy and early deaths.

Esophageal elongation techniques as Kimura extra-thoracic esophageal elongation, or Foker intra-thoracic traction and growth techniques, were used in cases of LGEA following the algorithm as previously described (10).

### Statistical Analysis

Period prevalence, Fisher exact test and Mann-Whitney test were used as appropriate. Results were expressed as mean and interquartile range. *P* < 0.05 was considered significant.

The statistical package used was SPSS v.17.0 for Windows.

## Results

During the study period 207 consecutive patients were admitted to our Department for EA/TEF. Since no preoperative deaths was observed, all newborn infants treated for EA/TEF underwent p-TBS, therefore entering in the present study.

### LTA Prevalence and Its Characterization

LTA period prevalence was 40.1%: 83 infants were affected by airways abnormalities (Table 1), while 124 did not experienced any LTA anomalies.

**Table 1:** LTA distribution.

	**Number of patients**	**%**
Overall	83	100
Tracehomalacia	53	64
Subglottic stenosis	13	16
Congenital subglottic stenosis	9	11
Vocal cord paralysis	20	24
Congenital vocal cord paralysis	6	7
Trachea-bronchial hypoplasia	4	5
Laryngo-tracheo-esophageal cleft	3	3.6
Esophageal lung	1	1.2
Others	8	9.8

#### Tracheomalacia

Tracheomalacia was the most common finding, diagnosed in 63% (*n* = 53) of patients. Thirty-eight patients presented moderate while 15 patients presented severe tracheomalacia. Of the 53 patients, 1 required aortopexy for treatment, within the first 6 months of life.

#### Vocal Cord Paresis

Twenty of the 83 children (24%) were found to have issues with vocal fold mobility: 6 patients experienced vocal fold hypomobility, while 14 had examinations consistent with paresis. Bilateral vocal cord paresis was present in 6 patients (7%); Left-sided involvement was reported in 2 patients. The remaining six patients had unilateral right-sided involvement. Eleven infants with vocal cord impairment presented LGEA, of whom 8 referred after a failed repair attempt (7 with cervical esophagostomy). Interestingly, LGEA patients presented a significantly higher rate of vocal cord paresis in comparison with non-LGEA infants (11/28, 39% vs. 9/55, 16%; *p* 0.03). Overall, congenital vocal fold hypomobility was present in a minority of patients presenting this issue (6 patients−7%).

#### Subglottic Stenosis

Preoperative airway evaluation in the operating room revealed in 13 infants (16%) subglottic stenosis (9 idiopathic, 4 due to prolonged intubation). Seven (54%) had grade 1 stenosis (1–50% obstruction), one infant (8%) grade 2 stenosis (51–70% obstruction), and five patients (38%) grade 3 stenosis (71–99% obstruction). No patients were diagnosed with grade 4 stenosis or complete obstruction. Within the group of patients presenting subglottic stenosis, 7 children required surgical intervention. Two children needed balloon or bougie dilation, while 6 patients eventually underwent tracheostomy; 3 patients are waiting for open anterior laryngotracheal reconstruction. Two patients died from co-morbidities.

#### Laryngeal Cleft

Laryngeal cleft was diagnosed in 4.1% (*n* = 3) of the study population. In this group, 2 patients had clefts classified as grade 1, and 1 patient as grade 4. Infants with grade 1 clefts were repaired endoscopically after esophageal atresia correction. Conversely, type 4 cleft radically modified surgical planning: surgical open approach to repair tracheo-esophageal cleft was primarily performed, associated to distal FTE closure, tracheostomy, cervical esophagostomy, and feeding gastrostomy. Esophageal reconstruction was delayed, after complete resolution of the condition.

### LTA Impact on Early Post-operative Outcomes

No differences were noted between LTA patients and those without LTA problems in terms of gender, gestational age and birth weight. Prematurity was present in one third of patients (67/207, 32%) with a similar distribution between the two groups, with and without LTA. Esophageal atresia types (according to Gross classification) were equally represented within the groups, as well as long gap esophageal atresia, and rate of associated abnormalities. Trends toward significance were noted in term of referral patients' rate, and frequency of cervical esophagostomy with a slight prevalence in Group A. Interestingly, genetic abnormalities were more frequently encountered in LTA infants in comparison Group B patients (10/83, 12% vs. 5/124, 4%, *p* 0.05). No differences were noted in term of associated cardiac abnormalities (either overall and considering only major heart anomalies). Conotruncal defects (e.g., aortopulmonary window; double outlet left ventricle; double outlet right ventricle), hypoplastic left heart syndrome, and valvular defects, were considered major cardiac defects. Table 2 summarized demographics and clinical characteristics at the time of first hospital admission.

**Table 2:** Demographic and clinical characteristics.

	**Overall 207 pts**	**Group A LTA + 83 pts**	**Group B LTA - 124 pts**	***P***
Gender; Male/Female	129/78	49/34	79/44	0.53
GA; weeks, median (IQR)	37 (34.25–39)	37 (34–39)	38 (35–39)	0.25
BW; gr, median (IQR)	2,500 (1,993–2,973)	2,400 (2,000–2,960)	2,565 (1,900–2,980)	0.32
Prematurity (< 37 weeks GA), *n* (%)	67 (32)	29 (35)	39 (31)	0.65
EA Type A-B/C-D-E	27/145	8/75	23/101	0.11
Referral, *n* (%)	39 (19)	21 (25)	18 (15)	0.06
Cervical esophagostomy (referral), *n* (%)	21 (10)	13 (16)	9 (7)	0.06
Long-gap EA, *n* (%)	63 (30)	28 (34)	35 (28)	0.44
Associated anomalies (other than LTA), *n* (%)	60 (29)	26 (31)	34 (27)	0.63
Genetic anomalies, *n* (%)	15 (7)	10 (12)	5 (4)	0.05
Congenital heart abnormalities	111 (53)	46 (55)	65 (52)	0.77
Major congenital heart abnormalities	17 (8)	8 (10)	9 (7)	0.61

Moreover, we found no differences between groups A and B concerning age at definitive esophageal repair, rate of primary esophageal anastomosis, need for traction and growth techniques, and re-do esophageal surgery. To note, Group A required tracheostomy more frequently (16/83, 19% vs. 0/124, *p* 0.0001), and presented a higher mortality rate (12/83, 14% vs. 5/124, 4%, *p* 0.009). Table 3 summarized main results of LTA on early outcomes.

**Table 3:** Surgical and clinical post-operative outcomes.

	**Overall 207 pts**	**Group A LTA + 83 pts**	**Group B LTA - 124 pts**	**p**
Age @ definitive esophageal surgery, mean (IQR)	4 (3–53)	4 (3–71.5)	3 (2–49)	0.37
Primary anastomosis, *n* (%)	163 (78)	61 (73)	102 (82)	0.16
Traction and growth techniques, *n* (%)	23 (11)	9 (11)	14 (11)	1.0
Re-do esophageal surgery, *n* (%)	26 (13)	14 (17)	12 (10)	0.14
Tracheostomy, *n* (%)	16 (8)	16 (19)	-	0.0001
Deaths, *n* (%)	17 (8)	12 (14)	5 (4)	0.009

## Discussion

This study demonstrates that patients treated for EA/TEF may frequently present upper airways' malformations, often clinically relevant. In the present cohort, the prevalence of LTA associated to EA/TEF was 40.5%, comparable with that reported in other series (5, 6, 11, 12).

To our knowledge this is the first prospective, and largest study exploring the association between LTA and EA/TEF infants. This is particularly important to focus the extension of this low reported association: although EA/TEF is an extensively studied condition, only few authors focused their attention on airway comorbidities (5, 11, 12). Conversely, association with other abnormalities such as chromosomal and genetic syndrome, associations or multiple anomalies without a pattern were repeatedly highlighted (2). This seems particularly strange, considering the common embryological origin of the lung bud (ventral respiratory diverticulum of the foregut) and the esophagus (from the dorsal portion of the foregut). Tracheoesophageal septum and sonic hedgehog are also involved in this initial foregut partition (13).

Remarkably, in our prospective cohort including all patients observed/referred for EA, the LTA prevalence was higher than many other major abnormalities, excluding major cardiac malformations, becoming the second most frequent comorbidity in EA/TEF population. This finding is consistent with others recently published papers (5, 6), highlighting the need for a preoperative complete airway examination in patients with EA/TEF. Given the rates of LTA, treatment and management decisions may be altered. Therefore, a collaborative approach between pediatric surgery and otolaryngology is warranted to optimize diagnosis and management for possible surgical treatment of upper respiratory tract anomalies.

Interestingly, two international surveys from the European Pediatric Surgeons' Association and the International Pediatric Endosurgery Group, recently found that preoperative bronchoscopy, in EA/TEF infants, was routinely performed only by 43% and 60% of responders, respectively, (79/178 and 102/170 responders) (14, 15).

Flexible laryngo-tracheo-bronchoscopy is an easy, fast and safe procedure to rule out possible LTA in newborn-infants. As a consequence, the preoperative evaluation of upper airway conditions in all EA/TEF infants is mandatory, since a delay in diagnosis (or a misdiagnosis) may led to increased early and late morbidity and mortality in the population, as well as possible legal sequelae. Our management protocol establishes, when an airway malformation is suspected during flexible laryngo-tracheo-bronchoscopy, the completion of the ENT evaluation using a rigid endoscopy to complete airway assessment.

Moreover, preoperative flexible laryngo-tracheo-bronchoscopy performed at the time of esophageal surgery, reduces multiple exposures to anesthesia in fragile patients, limiting the possible reported detrimental effects on lasting deficits in learning and cognitive processes (16).

Moderate to severe tracheomalacia was the most frequent findings in our LTA patients, with prevalence above 60% (51/83 patients) (Table 1). Similarly, other studies confirmed a high rate of tracheomalacia in EA/TEF infants, ranging between 37 and above 80% (5, 6, 17). Nonetheless, diagnosis of tracheomalacia is still debated: it is usually considered a co-morbidity in EA/TEF infants, and most of the symptoms at onset are not specific. Hence, it is mandatory to rule out the presence of this condition before esophageal correction, in order to limit diagnostic biases. In suspected cases, clinically relevant tracheomalacia, radiographic (dynamic CT chest scan) and endoscopic evaluations are necessary to confirm the diagnosis: tracheomalacia is commonly outlined in cases of airway collapse associated with severe airway symptoms, either idiopathic or secondary to other conditions. Therefore, there is a need for waiving other possible etiologies of airway compromise such as associated cardiac or vascular malformations, gastro esophageal reflux, and esophageal stricture (6, 17–19).

The impact of tracheomalacia on outcome is well documented (6, 18, 19), with a need for surgical correction variably reported (6, 17) between 2 and 36% of cases. The classical approach to symptomatic tracheomalacia is represent by aortopexy, either performed by open surgery or thoracoscopically (19). Nonetheless, some authors recently proposed posterior tracheopexy when tracheomalacia is sustained by posterior intrusion of “pars membranacea” (18). Furthermore, Shieh and coworkers suggested to perform tracheopexy at the time of initial EA/TEF repair to effective treat severe tracheomalacia with significant improvement or resolution of clinical symptoms and degree of airway collapse on bronchoscopy (20).

Although this approach seems to be successful, the risk of overtreatment has to be considered when preventive tracheopexy is performed: generally, tracheomalacia spontaneously resolved in more than 80% of EA/TEF patients (6, 17).

In our experience, patients presenting severe tracheomalacia did not require surgical correction unless tracheomalacia was associated to other major cardiac, or vascular, anomalies. Those, causing compression on the trachea, ultimately produced tracheomalacia: 3 out 83 (4%) LTA patients in our cohort, associated to cardiac surgery (2 patients) or to major esophago-tracheal surgery (1 patient with grade IV laryngo-tracheo-esophageal cleft) ultimately required surgery for tracheomalacia, in all cases.

In our population, demographic and clinical characteristics did not differ between patients with or without LTA; a trend toward statistical significance was observed concerning patients referred from other centers (in most cases after a previous failed attempt of esophageal correction) and the rate of cervical esophagostomy (also performed in referring centers) (both *p* 0.06). Previously, ourselves and other authors, have demonstrated that referral EA/TEF patients presented an increased risk for post-operative complications (10, 21). The presence of LTA may further increase the overall management complexity of those patients: we did not found differences between Group A and Group B patients in term of age at definitive esophageal correction, rate of primary anastomosis, or need for a redo esophageal surgery. Nonetheless, LTA patients presented a higher prevalence of tracheostomy formation mainly due to severe subglottic stenosis (7 /16, 44%) or vocal cord paralysis (5/16, 30%). Tracheotomy was offered to provide life-sustaining airway support for those children with acute or chronic airway obstruction, and pulmonary insufficiency, thereby circumventing anatomical and physiologic obstruction to ventilation. Moreover, our series showed an increased mortality rate associated to the presence of airway anomalies.

As a consequence, we strongly encourage a multidisciplinary approach, as previously suggested (3) even for initial assessment to assure the best surgical approach to EA and, even more critical, to prevent late sequelae. Specifically, we believe that the high prevalence of LTA in EA/TEF infants, and the increasing awareness of associated increase of morbidity and mortality, strongly suggest spreading use of preoperative bronchoscopy as a standard procedure.

In conclusion, airways abnormalities are frequently encountered in EA/TEF infants, becoming the most frequent ones in those patients, after cardiac anomalies. Our data suggest that the presence of LTA may represent a risk factor of early morbidity and mortality preventable with early diagnosis and pro-active management. Systematic endoscopic preoperative evaluation has to be pushed forward to minimize LTA-related morbidity and mortality, and to coordinate airway management along with EA/TEF procedures.

## Author Contributions

AC, LV, MS, and PB: study conception and design. AC, LV, and MS: data acquisition. AC, LV, MS, MT, DM, SS, SB, and PB: analysis and data interpretation. AC: drafting of the manuscript. AC, LV, MS, MT, DM, SS, SB, and PB: critical revision.

### Conflict of Interest Statement

The authors declare that the research was conducted in the absence of any commercial or financial relationships that could be construed as a potential conflict of interest.
